# Movement reveals reproductive tactics in male elephants

**DOI:** 10.1111/1365-2656.13035

**Published:** 2019-06-24

**Authors:** Lucy A. Taylor, Fritz Vollrath, Ben Lambert, Daniel Lunn, Iain Douglas‐Hamilton, George Wittemyer

**Affiliations:** ^1^ Department of Zoology University of Oxford Oxford UK; ^2^ Save the Elephants Nairobi Kenya; ^3^ Department of Infectious Disease Epidemiology Imperial College London London UK; ^4^ Department of Statistics University of Oxford Oxford UK; ^5^ Department of Fish, Wildlife, and Conservation Biology Colorado State University Fort Collins Colorado

**Keywords:** bio‐logging, elephant, GPS, movement, musth, reproduction

## Abstract

1. Long‐term bio‐logging has the potential to reveal how movements, and hence life‐history trade‐offs, vary over a lifetime. Reproductive tactics in particular may vary as individuals' trade‐off current investment versus lifetime fitness. Male African savanna elephants (*Loxodona africana*) provide a telling example of balancing body growth with reproductive fitness due to the combination of indeterminate growth and strongly delineated periods of sexual activity (musth), which results in reproductive tactics that alter with age.
2. Our study aims to quantify the extent to which male elephants alter their movement patterns, and hence energetic allocation, in relation to (a) reproductive state and (b) age, and (c) to determine whether musth periods can be detected directly from GPS tracking data.
3. We used a combination of GPS tracking data and visual observations of 25 male elephants ranging in age from 20 to 52 years to examine the influence of reproductive state and age on movement. We then used a three‐state hidden Markov model (HMM) to detect musth behaviour in a subset of sequential tracking data.
4. Our results demonstrate that male elephants increased their daily mean speed and range size with age and in musth. Furthermore, non‐musth speed decreased with age, presumably reflecting a shift towards energy acquisition during non‐musth. Thus, despite similar speeds and marginally larger ranges between reproductive states at age 20, by age 50, males were travelling 2.0 times faster in a 3.5 times larger area in musth relative to non‐musth. The distinctiveness of musth periods over age 35 meant the three‐state HMM could automatically detect musth movement with high sensitivity and specificity, but could not for the younger age class.
5. We show that male elephants increased their energetic allocation into reproduction with age as the probability of reproductive success increases. Given that older male elephants tend to be both the target of legal trophy hunting and illegal poaching, man‐made interference could drive fundamental changes in elephant reproductive tactics. Bio‐logging, as our study reveals, has the potential both to quantify mature elephant reproductive tactics remotely and to be used to institute proactive management strategies around the reproductive behaviour of this charismatic keystone species.

## Graphical abstract

Combining GPS tracking with visual observations of male elephants, the authors found that older individuals markedly increased their daily speed and range use when reproductively active. Reproductively associated movements were not identifiable for younger individuals. Automatic detection of reproductive state in mature male elephants can be accomplished with GPS tracking.

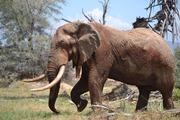

## 1 INTRODUCTION

Advances in bio‐logging technology have revolutionized the study of animal movements by enabling the remote, quantitative study of movement, which increases the range of potential study species, enables research in inaccessible habitats, removes observer bias and, most importantly, means that data can be gathered on individuals out of sight and sound of the researcher (Kays, Crofoot, Jetz, & Wikelski, 2015; Nathan, 2008; Nathan & Giuggioli, 2013; Westley, Berdahl, Torney, & Biro, 2018). As bio‐logging technology continues to advance, our ability to answer both exciting new questions and to explore fundamental theory and life‐history trade‐offs in depth increases (Nathan et al., 2008). Moreover, advances in on‐board processing may lead to many of these behaviours being monitored in real time, which can be used in both research and proactive conservation and management strategies (Wall, Wittemyer, Klinkenberg, & Douglas‐Hamilton, 2014). A major challenge still remaining is the long‐term monitoring of individual movements, which is currently limited by ethical, logistical and device constraints, such as battery life (Goldenberg, Douglas‐Hamilton, & Wittemyer, 2018; Shillinger et al., 2012). As optimal decisions are likely to vary over the life span of an individual, long‐term bio‐logging has the potential to reveal new insights into how movements, and hence life‐history trade‐offs, vary over a lifetime (Stearns, 1992).

In polygynous mating systems, variation in reproductive success is greater in males than females (Andersson, 1994; Clutton‐Brock, Albon, & Guinness, 1989; Emlen & Oring, 1977). One male may mate with a number of females, thereby increasing the reproductive success of that individual. The resultant selective pressure from male–male competition for access to females (intrasexual selection) and/or female mate choice (intersexual selection) has been suggested to be the driving force behind the evolution of exaggerated traits (Andersson, 1982, 1994; Darwin, 1871; Emlen & Oring, 1977; Kodric‐Brown & Brown, 1984). Intrasexual competition, in particular, can lead to the evolution and escalation of traits which are advantageous in conflict situations, such as increased size and physical weapons (Andersson, 1994; Darwin, 1871). Based on the assumption of limited resources, life‐history theory posits the importance of trading‐off between certain traits, such as current reproductive effort versus survival, growth and future fecundity (Stearns, 1992). Not surprisingly, investing significant resources into reproduction can lead to a shorter life expectancy (Andersson, 1994). Thus, males may adopt different reproductive tactics according to their relative condition, strength and experience, which strongly influence their potential to reproduce successfully. As energy is a finite resource, analysing how an individual allocates its energy resources, such as through movement, can help us to identify different reproductive tactics.

The largest land mammal, the African savanna elephant (*Loxodonta africana*), provides a telling example for the dilemma of balancing energetic investment in both body size and reproduction. The combination of longevity, indeterminate growth and sexual dimorphism means that old males can reach over twice the body mass of both females and young males (Laws, 1966). Male elephants are physiologically able to reproduce by 14–17 years old giving them a potential reproductive life span of over 40 years, although younger males are often competitively excluded from active reproduction (Hollister‐Smith et al., 2007; Rasmussen, 2005; Rasmussen, Okello, et al., 2008). Beyond age ~25, males start to display periods of heightened sexual and aggressive activity termed musth, which is likened to rutting behaviour in deer (Ganswindt, Rasmussen, Heistermann, & Hodges, 2005; Poole & Moss, 1981). Musth is a powerful trait that can temporarily raise the dominance status of an individual to above those not in musth (Poole, 1987, 1989a). Musth duration and regularity is positively related to age, with males over 35 displaying regular musth periods once a year for an extended duration (~2 months), whilst younger bulls between ~26 and 35 tend to express musth characteristics for up to 2 weeks several times a year (Poole, 1987; Poole, Lee, Njiraini, & Moss, 2011). Thus, the reproductive success of a male elephant increases with age, both through reductions in intrasexual competition by the escalation of size‐related dominance (Poole, 1989a), and an increase in intersexual selection by females for older males (Moss, 1983; Poole, 1989b). However, despite the reproductive dominance of older males, younger males still contribute to the gene pool (Hollister‐Smith et al., 2007; Rasmussen, Okello, et al., 2008). African savanna elephants, henceforth referred to as elephants, are thus an ideal species to examine variation in reproductive tactics over time due to their distinctive reproductive periods, indeterminate‐growth structured life history and their physical size, which means they are amenable to carry bio‐loggers for long periods of time with little impact. Moreover, investigating how elephant reproductive tactics, and hence allocation to life‐history traits, vary with age is crucial to our understanding of the behavioural ecology of the African savanna elephant and, ultimately, the driving forces shaping the evolution of their life history.

Our study aimed to use a combination of bio‐loggers and physiologically based visual assessment of reproductive state (Ganswindt et al., 2010, 2005; Rasmussen, Ganswindt, Douglas‐Hamilton, & Vollrath, 2008) to answer fundamental behavioural questions about elephant reproductive behaviour. In particular, we aimed to (a) quantify the extent to which male elephants alter their movement patterns, and hence allocate their energy, between musth and non‐musth periods, (b) investigate whether reproductively structured movement patterns change with age (where age is a correlate of body size and dominance rank) and (c) determine whether musth periods can be detected directly from GPS tracking data. We hypothesized that males in musth would increase their movement patterns relative to non‐musth reflecting an increase in searching effort for receptive females. Given that older males display distinctive musth periods, whereas younger males tend to be more opportunistic in their mating tactics (Poole, 1989a; Poole et al., 2011; Rasmussen, 2005; Rasmussen, Okello, et al., 2008), we hypothesized that the contrast between musth and non‐musth movements would be larger in older males. The ability to detect musth via GPS tracking data could enable the remote study of key life‐history parameters as well as the ability to institute proactive management strategies around males in this state.

## 2 MATERIALS AND METHODS

### 2.1 Subjects

The study population of elephants inhabits the semi‐arid region in and around the unfenced Samburu and Buffalo Springs National Reserves in northern Kenya (0.3–0.8°N, 37–38°E). The area is dominated by *Acacia–Commiphora* savanna and scrub bush along the semi‐permanent Ewaso N'giro River, which is the major water source in the region. Rainfall averages approximately 350 mm per year and occurs biannually during April–May and November–December (Wittemyer, 2001). The study population has been monitored since 1997 and consists of ~900 individuals, of which ~18% are adult bulls and the remainder are breeding females and their calves that can all be individually identified using ear and tusk idiosyncrasies (Rasmussen, Okello, et al., 2008; Wittemyer, 2001). All adult individuals have been aged to the nearest year during the period of data collection, based on physical appearance using individuals aged from molar progression as reference points (Rasmussen, Wittemyer, & Douglas‐Hamilton, 2005). All males were assigned the birth date of the 31st August, which is the mid‐point of a 4‐month period during the dry season when few bulls were in musth and meant that bulls did not change age category during the peak musth periods November–February and April–June (Table S1).

### 2.2 Movement data

GPS tracking collars were fitted to 30 adult bull elephants inhabiting Samburu and Buffalo Springs National Reserves. Of these 30 bulls, 25 bulls were observed either in and/or out of musth with a GPS tracking frequency of 1‐hr intervals or less, with the remainder having tracking dataset with less frequent location collection. The collars were fitted by a veterinarian from the Kenya Wildlife Service (KWS) following national protocols on animal handling. To maintain consistency, the ten collars from eight different bulls with all or some 15 or 30‐min fixes were systematically down‐sampled to 1‐hr sampling frequencies on the hour. A continuous‐time correlated random walk model was fitted to predict temporally regular locations for missing data points ≤4 hr (Johnson, London, Lea, & Durban, 2008). Days with data gaps >4 hr were excluded from the analysis. For each GPS point, the orthodromic (great‐circular) distance from the previous point was calculated using the haversine formula. We calculated the daily mean speed (km/hr) as an analogue for distance using the distance over the time between each GPS fix, which enabled us to include days with a small amount of missing GPS fixes (≥20 samples). Daily range size was estimated using a 95% Minimum Convex Polygon (MCP) (Mohr, 1947). Given we are estimating daily range size (i.e. 24 GPS points), we selected MCP as a simple, assumption free means by which to estimate space use. Using 100% MCP resulted in approximately the same level of musth detection, so we present the more conservative daily range size using the 95% MCP. We also calculated the sinuosity index proposed by Benhamou (2004), but sinuosity index was highly correlated with speed and 95% MCP in this study (Figure S1), so we only present the results for speed and 95% MCP.

### 2.3 Musth observations

All elephants encountered during regular patrols of Samburu and Buffalo Springs National Reserves have been recorded since 1997, including the identity of the individual(s) present, date and time, location and notes on reproductive behaviour. For males, the degree of temporal gland swelling, temporal gland secretion and urine dribbling were recorded following criteria outlined by Poole (1987). We analysed the data using musth as a categorical covariate as either musth or non‐musth. To be considered as in musth, the bulls had to display 2–3 musth signals. The endocrine underpinnings of these signals were assessed in the study ecosystem previously, demonstrating the visual scoring used in this study is indicative of male reproductive hormone levels (Ganswindt et al., 2010, 2005; Rasmussen, Ganswindt, et al., 2008). Observations where bulls displayed just one signal, such as temporal gland secretion, were excluded from the analysis (53 observations). We assumed that the bull was in the same state both the day before and the day after a field observation. In total, we compared 1,375 non‐musth days to 496 musth days from bulls ranging in age from 20 to 52 at the time of observation (Table 1).

**Table 1:** Sample information including minimum and maximum age and the number of non‐musth and musth days analysed for each bull. The observations include all days where the bull was observed with ≥20 GPS fixes. The total GPS tracking days analysed includes one day either side of the day the bull was observed. Note for some observations, GPS tracking data were missing for the date of the observation itself, but data were available from the days either side (21 musth observations and 18 non‐musth observations). Individuals are ordered by the maximum age attained during the study period

Bull name	Age (years)		Observations with GPS tracking data		Total GPS tracking days analysed	
	Min	Max	Non‐musth	Musth	Non‐musth	Musth
Bahati/Nusura	20	20	2	1	4	3
Kiir	21	22	3	1	9	3
Ansel	22	24	25	0	60	0
Columbus	26	27	35	0	86	0
Lemaiyan	27	27	1	1	3	3
Nehru	28	29	9	1	25	3
Thoreau	23	31	5	1	13	3
Picasso	31	32	9	3	25	5
Edison	29	33	7	17	19	37
Frank	33	33	1	8	2	21
Uffe	21	34	28	3	77	14
Winston	28	34	89	19	221	47
Theresai	33	34	7	3	20	7
Boru	32	37	4	1	14	3
Nelson Mandela	36	37	12	0	28	0
MLK	37	37	2	3	4	10
Apollo	36	38	56	20	131	51
Boone	39	39	3	6	9	13
Leakey	40	41	2	0	3	2
Lewis	41	41	7	0	18	0
Esidai	35	44	80	30	217	75
Kenyatta	42	44	52	21	136	44
PrettyBomBom	44	45	33	32	92	80
Mungu	46	48	64	22	153	51
Matt	39	52	2	6	6	21
Total			538	199	1,375	496

### 2.4 Environmental covariates

Movement patterns of elephants have been shown to be affected by vegetation productivity, terrain slope and ruggedness, distance to the nearest water source and whether the elephant is in or out of a protected area (Boettiger et al., 2011; Bohrer, Beck, Ngene, Skidmore, & Douglas‐Hamilton, 2014; Ihwagi et al., 2018; Wall, Douglas‐Hamilton, & Vollrath, 2006; Wittemyer, Polansky, Douglas‐Hamilton, & Getz, 2008). Therefore, we included these environmental factors as covariates for each GPS point. We used NASA's Terra Moderate Resolution Imaging Spectroradiometer (MODIS) 16‐day 250 m version 6 composites of normalized difference vegetation index (NDVI) to measure time‐specific vegetation productivity (Didan, 2015). Slope (0–90°) and vector ruggedness measure (VRM; Sappington, Longshore, & Thompson, 2007) were calculated using the Shuttle Radar Topography Mission (SRTM) 30 m V3 elevation dataset using the eight neighbouring cells (Farr et al., 2007). Distance to water (km) was estimated as the distance to nearest permanent rivers, lakes or swamps. A binary covariate was added to distinguish whether an elephant was in or out of a protected area. The NDVI and elevation datasets were downloaded from Google Earth Engine. Maps of protected areas and water sources were developed in‐house by Save the Elephants.

### 2.5 Influence of reproductive state and age on movement patterns

We analysed daily mean speed and 95% MCP using linear mixed‐effects (LME) models. Both daily mean speed and 95% MCP were positively skewed and were therefore log‐transformed prior to analysis. The fixed effects consisted of musth state as a binary covariate interacting with age at observation (years), age^2^, daily mean NDVI, mean NDVI^2^, mean slope, mean slope^2^, mean VRM, mean VRM^2^, mean distance to water, mean distance to water^2^ and protected area (Figure S2). In order to avoid possible computer rounding errors and confine coefficient estimates to a reasonable scale, all continuous environmental covariates were standardized by subtracting the mean and dividing by the standard deviation $\left(\frac{x-\overset{¯}{x}}{\sigma }\right)$ prior to analysis. Age was kept in years and was centred on age 35, which is the age bulls start to exhibit clear musth periods (Poole, 1987; Poole et al., 2011), to aid in interpretation. Protected area was rounded to the nearest binary variable as either in (1) or out (0) of a protected area. Log‐likelihood tests revealed that the elephants responded differently to NDVI (i.e. log‐transformed daily mean speed likelihood‐ratio test [LRT]: *p < *0.001), and therefore, the random effect of elephant identification was added on both the intercept and NDVI slope. We added two different within‐group correlation structures. Firstly, whilst homoscedasticity was found within both musth state and non‐musth state, their variances were not the same, and therefore, a weighting factor was added to the model to cater for this distinction. Secondly, we added an autoregressive lag‐1 correlation (corAR1) to the models to account for the temporal relatedness of observations, which proved necessary when we tested the model with and without the corAR1 using a likelihood‐ratio test (LRT) (Pinheiro & Bates, 2000) (Figure S3).

Linear mixed‐effects models were fitted with the R package “nlme” (Pinheiro, Bates, DebRoy, Sarkar, & R Core Team, 2018). From the full models, parsimonious models were obtained by deletion of non‐significant terms using LRTs. LRTs were used to test the statistical significance of each fixed effect in the best‐fitting model. The models were then refitted using restricted maximum likelihood to obtain robust parameter estimates and confidence intervals. Final models were checked for assumptions of linearity, normality, homoscedasticity and autocorrelation by visual inspection of plotted residuals and autocorrelations.

Data processing and analysis was conducted in the open‐source software R (version 3.4.3, R Core Team, 2017), using the packages adehabitatHR (version 0.4.15, Calenge, 2006), MASS (version 7.3‐48, Venables & Ripley, 2002), MomentuHMM (version 1.4.0, McClintock & Michelot, 2018), nlme (version 3.1‐131.1, Pinheiro et al., 2018) raster (version 2.6‐7, Hijmans, 2017), rgdal (version 1.2‐16, Bivand, Keitt, & Rowlingson, 2017), rgeos (version 0.3‐26, Bivand & Rundel, 2017), sp (Bivand, Pebesma, & Gómez‐Rubio, 2008; version 1.2‐7, Pebesma & Bivand, 2005) and spatialEco (version 1.0‐1, Evans, 2017).

### 2.6 Musth detection

To determine whether musth can be detected from GPS data, we analysed the data using a three‐state hidden Markov model (HMM), which detects distinct changes in movement. The HMM priors were based on the estimates from the linear mixed‐effects models (although allowed considerable freedom to allow the state‐specific parameters to be determined), which showed that bulls increased their movement patterns in musth. Thus, the three states comprised a slower speed at the start of the selected time period (State 1), followed by an increase in speed in State 2 and a decrease in speed in State 3. The three latent states were ordered, so no other sequence of moves was possible. We modelled the log‐transformed daily mean speed and log‐transformed 95% MCP of the elephants using a normal sampling model whose parameters depended on the estimated state of the elephant, and thus, the emission of the log‐transformed mean speed ($\overset{¯}{S}$) at time (*t*) followed:

$${\overset{¯}{S}}_{t}|k∼\text{normal}\left(\beta_{k},\;\sigma_{k}\right)$$

where *k* is the latent state, *β_k_* is the mean parameter in that state, and *σ_k_* is the standard deviation. We did not include any environmental covariates in the three‐state hidden Markov models to test whether musth signals can be directly detected from GPS tracking data alone, which is important for the applicability of the method for management purposes. The model priors can be found in Table S2. We conducted posterior predictive checks of the model fit, which indicated that there was autocorrelation in the predicted step lengths. Models including an autoregressive error term, however, worsened our ability to estimate musth relative to a model without this correction (presumably because the former overly smooths the data). Our results, therefore, are for the model with independent errors. Other posterior predictive checks indicated that the means and maxima step lengths were well replicated by the model. Finally, pp plots of the theoretical versus empirical cumulative distributions (Figure S4) indicated that the stepping distribution used (a normal) was a reasonable fit to the data. Three‐state HMMs were written in Stan (Carpenter et al., 2017) using the R interface RStan (version 2.17.3, Stan Development Team, 2017). The HMM code and an example model output are available on the Dryad Digital Repository (Taylor et al., 2019).

To test whether the HMM could detect musth periods, we analysed a subset of our dataset containing sequential GPS tracking data of 15 musth periods from 10 male elephants, ranging in age from 28 to 52 years, that provided the best combination of field observations with sequential tracking data over an entire season. Each bull had a minimum of six visual observations, including at least one observation when the bull was in musth, and a minimum of 120 days of corresponding GPS tracking data with <10% missing data. Where possible, we took 120 days either side of the mid‐point of the musth observations. Eight Markov chains were run simultaneously, each with 5,000 warm‐up and 5,000 sampling iterations, which resulted in 40,000 samples for each posterior distribution. We diagnosed convergence in the sampling distribution as determined by using a criterion $\hat{R}⩽1.1$ across all parameters (Gelman & Rubin, 1992). To test the sensitivity and specificity of the three‐state HMM, we examined whether the states detected correctly corresponded to the visual observations of the bulls in musth or non‐musth. We calculated the standard deviation in the estimated duration of musth from the posterior samples.

## 3 RESULTS

### 3.1 Influence of reproductive state and age on movement patterns

**Figure 1:**
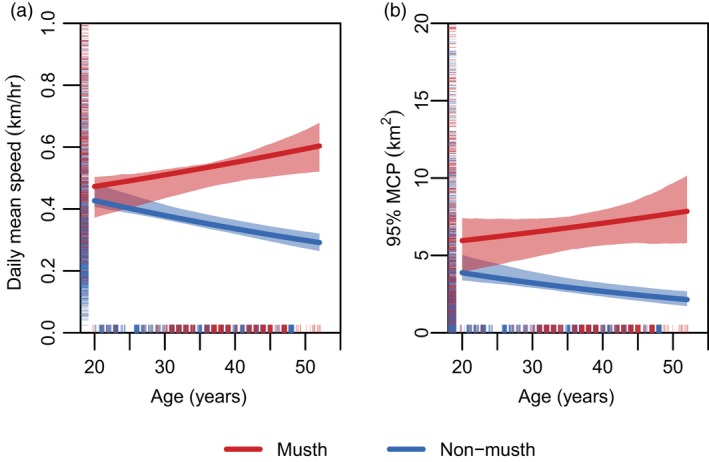
Estimated relationship between (a) daily mean speed (km/hr) and (b) 95% MCP (km^2^), and age (years) in musth (red) and non‐musth (blue). Shaded areas correspond to the 95% confidence interval from a nonparametric bootstrap of 1,000 resampled data points. The raw data are illustrated by the points on the axes. A scatter plot of the raw data can be found in Figure S5

The results of our analysis revealed that reproductive state had a statistically significant effect on both daily mean speed and range size (daily 95% MCP). Daily mean speed increased 1.49 times (log‐transformed daily mean speed likelihood‐ratio test: $\chi_{1}^{2}$ = 67.07, *p < *0.001) and 95% MCP 2.31 times ($\chi_{1}^{2}$ = 71.4, *p < *0.001) in musth relative to non‐musth for a bull aged 35 years under average environmental conditions (Table S3). Furthermore, speed diverged with age, with bulls both decreasing their non‐musth speed with age ($\chi_{1}^{2}$ = 5.36, *p = *0.021), and increasing their musth speed with age ($\chi_{1}^{2}$ = 11.82, *p < *0.001; Figure 1a). Thus, at age 20, there was no detectable difference between musth and non‐musth daily mean speed ($\chi_{1}^{2}$ = 0.95, *p = *0.330), but, by age 50, males were travelling 1.99 times faster in musth than non‐musth ($\chi_{1}^{2}$ = 64.39, *p < *0.001). Range size (95% MCP) in musth also increased with age ($\chi_{1}^{2}$ = 5.60, *p = *0.018), whereas non‐musth range size decreased but not significantly with age ($\chi_{1}^{2}$ = 3.14, *p = *0.076; Figure 1b). Moreover, unlike speed, the range size of a 20‐year‐old male was 1.54 times larger in musth than non‐musth ($\chi_{1}^{2}$ = 3.93, *p = *0.047). By age 50, the range size of a male in musth was 3.47 times larger than in non‐musth ($\chi_{1}^{2}$ = 51.79, *p < *0.001).

### 3.2 Musth detection

**Figure 2:**
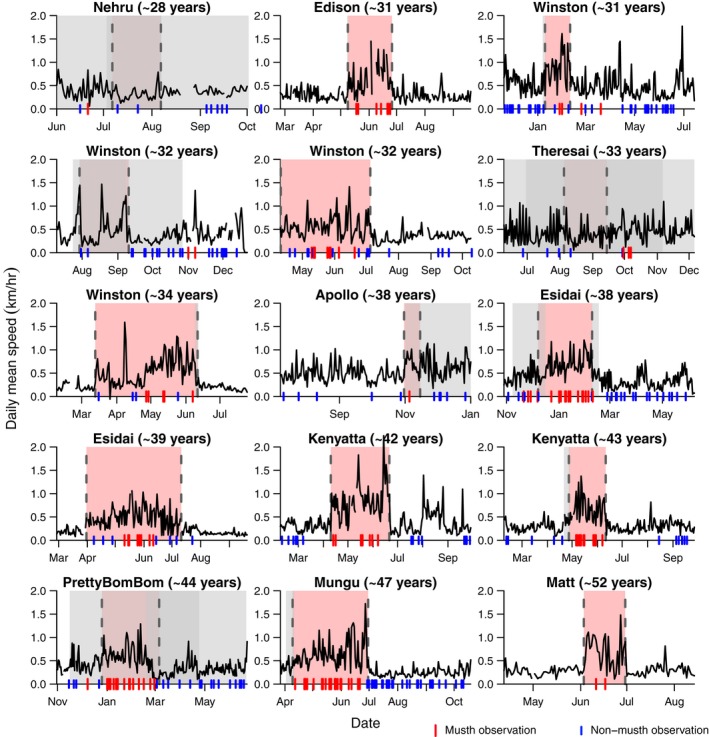
Three‐state hidden Markov model results of the model for log‐transformed daily mean speed aiming to detect musth periods in bull elephants. Plots show the untransformed daily mean speed with the detected musth periods shaded in red. Grey shaded area indicates the corresponding credible interval (±95%). Visual observations of the bull in musth or non‐musth are denoted by the red and blue lines at the base of the plot. Plots are ordered by age from youngest to oldest

Overall, the fixed three‐state HMM of log‐transformed daily mean speed detected musth periods which resulted in 90% of all observations being correctly identified (musth or non‐musth) across the 10 bulls studied. A total of 106 musth observations were correctly assigned with eight false positives (84% sensitivity after accounting for the effects of individual; Table S4), and 162 non‐musth observations were correctly assigned with 39 false negatives (84% specificity). Aside from one bull, “Edison”, the models of bulls under the age of 35 tended to have a lower sensitivity (66%; true positive) and specificity (77%; true negative) compared with the bulls over 35, where the models had high sensitivity (97%) and specificity (88%) due to the greater differentiation between musth and non‐musth movements in older male elephants (Figure 2). Furthermore, the standard deviations in the estimated duration of musth were lower in the bulls over 35 (5.1 days compared with 18.3 days), indicating a higher degree of certainty in the timing of the detected state changes between musth and non‐musth in the older individuals.

**Figure 3:**
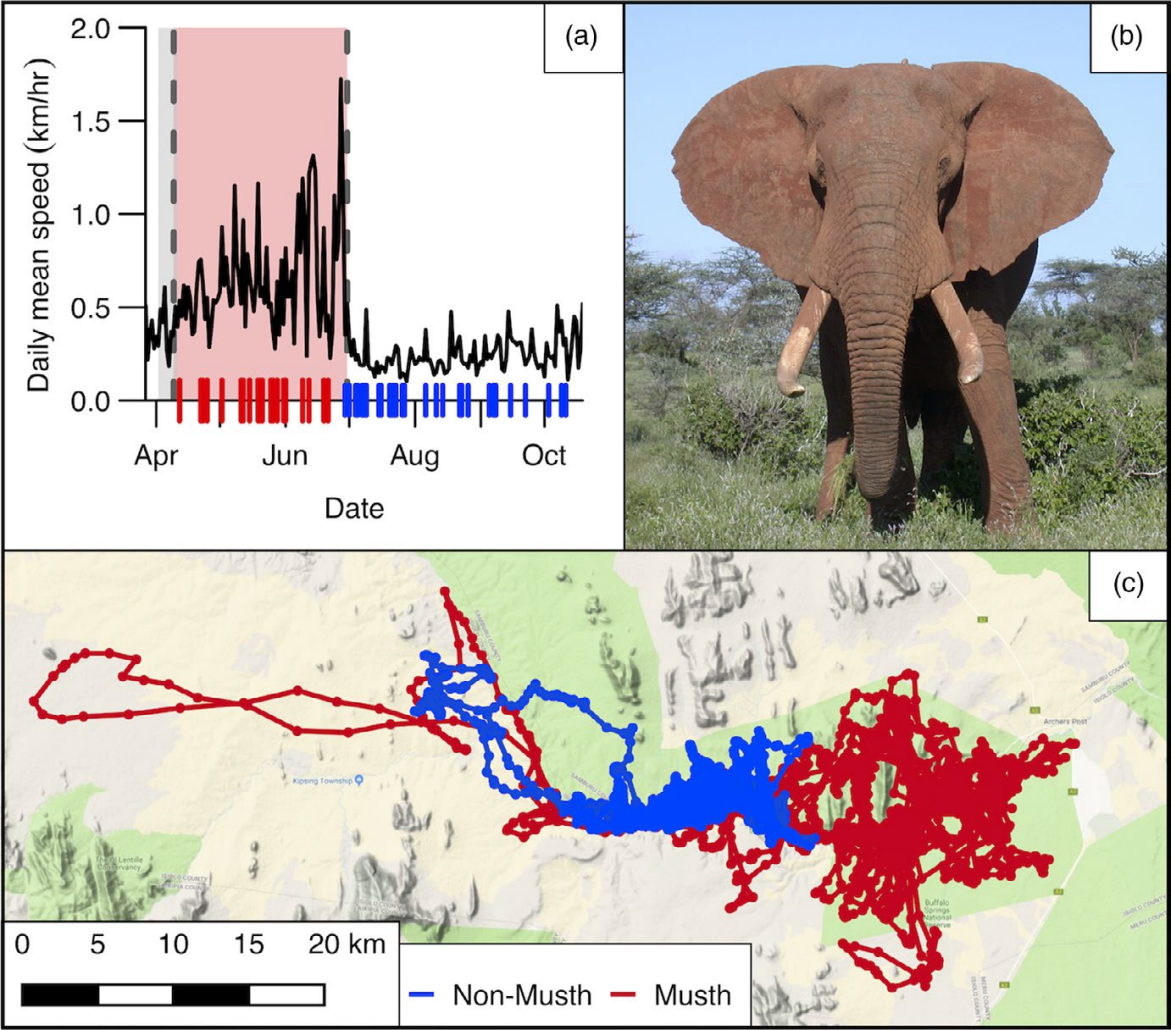
Detected musth and non‐musth periods for “Mungu” (B1001) aged ~47 years. (a) The untransformed daily mean speed and the assigned musth period (shaded red area) result from the three‐state hidden Markov model. Visual observations of the bull in musth or non‐musth are denoted by the red and blue lines at the base of the plot. (b) Photo: G. Wittemyer. (c) Map of the corresponding GPS tracking data for the detected musth (red lines) and non‐musth (blue lines) periods for Mungu

During the assigned musth periods, the daily mean speed of males over 35 was ~2.14 times faster than non‐musth periods (Figure 3). Furthermore, the standard deviation of speed during musth increased ~2.11 times, which probably relates to searching behaviour interspersed with periods of guarding receptive females. Males under 35 also increased their speed in musth, although interpretations are limited due to the lower sensitivity and specificity of the models. Given that speed and MCP are correlated, the results for the three‐state HMM for log‐transformed 95% MCP were similar to the speed results, but the models had slightly lower levels of sensitivity and specificity (Figure S6, Table S5). As such, automatic detection algorithms for musth state are likely to have higher performance when applied to speed of movement than use of area.

## 4 DISCUSSION

Determining the motivations underlying movement patterns remains a key objective in movement ecology research, but often it is challenged by limited insight to the context of locational data. We used a unique combination of locational data, characterizing both movement and space use, with physiologically informed observational data on reproductive state in male African savanna elephants, which enabled us to characterize contextualized movement behaviour in detail. Our results demonstrate that mature males increase their daily mean speed and range size when in musth (their reproductively active state). As locomotion is energetically expensive, our results demonstrate that musth comes at a considerable energetic cost to an elephant, in addition to costs associated with competitive interactions with other males. Moreover, the strong age effect on movement of bulls in musth suggests that the energetic allocation into reproduction also increases with age. The corresponding decrease in non‐musth speed with age likely reflected a singular shift towards energy acquisition during non‐musth among older males. The combined effect of musth and non‐musth movements meant that, despite similar speeds and marginally larger ranges between states at age 20, 50‐year‐old males were travelling twice as fast in an area that was three and a half times larger when in musth relative to non‐musth. This strong juxtaposition between musth and non‐musth movements allowed the accurate detection of musth periods in males over age 35 directly from movement data using a three‐state hidden Markov model. As such, bio‐logging alone has the potential to both remotely quantify mature elephant reproductive tactics and be used to institute proactive management strategies around the reproductive behaviour of this charismatic keystone species.

The increase in movements during musth is congruent with behavioural observations indicating that bulls spend more time walking and less time foraging and resting in musth, altering their behaviour to enhance the time spent searching for prospective mates (Hall‐Martin, 1987; Poole, 1989a). Given the relative rarity of reproductive events in the life of a female, resulting from their 22‐month gestation and multi‐year period of lactational dependence, and the large areas used by female savanna elephants (Lee & Moss, 2011; Loarie, Aarde, & Pimm, 2009; Wittemyer, Ganswindt, & Hodges, 2007), active searching is likely to be critical to male reproductive success. Thus, the increased probability of finding receptive females may outweigh the energetic cost for elephants due to the potential to increase an individuals' reproductive success.

Elephants demonstrate strong age‐related reproductive skew (Hollister‐Smith et al., 2007; Rasmussen, Okello, et al., 2008), and examining age differentiated movement patterns provides insight to the mechanisms driving this skew. Although male elephant body mass continues to grow throughout life, shoulder height asymptotes, which means there is not a strong allometry between leg length and walking speed (Hutchinson et al., 2006). Thus, young elephants can move as fast/efficiently as older elephants (Hutchinson et al., 2006), which means that differences in size are unlikely to drive the observed increase in musth movements with age. Moreover, alongside increases in speed, musth duration increases with age (Poole et al., 2011), which means older bulls are both moving faster and for longer portions of the year relative to younger males. Overall, older males are allocating energetic resources into musth periods, likely causing the elevated body condition declines observed among older bulls during musth (Poole, 1989a). In combination, these observations indicate that energy expenditure during musth exceeds energy acquisition. The substantial decrease in body condition during musth could be associated with the observed decrease in non‐musth speed with age, where older males focus on energy acquisition during non‐musth periods. Thus, the combined evidence of the extended duration of musth in older individuals (Poole et al., 2011), the greater degree of body mass loss in older individuals (Poole, 1989a) and the increase in movements during musth in relation to age found in this study suggest that the energetic allocation into locating receptive females in musth increases with age.

Elephants are unusual in that reproductive success peaks relatively late in life (Andersson, 1994). A key life‐history trait that appears to skew the reproductive success of male elephants towards older age is the trait of indeterminate growth in body mass, the rarity of which has limited the investigation of indeterminate growth on life‐history traits and reproductive tactics. As a result of indeterminate growth, the observed elevated energetic allocation in movements during musth periods by older males coincides with when they attain their highest lifetime dominance rank and when their residual life expectancy is low, which means males allocate more and may take more risks for gains in reproductive success. By contrast, in younger males, both the probability of reproductive success is lower and the residual life expectancy is higher, which means the proximate costs of movement may not outweigh the benefits gained through the increased potential of finding receptive females. As such, younger males appear to take on opportunistic reproductive tactics (Rasmussen, 2005), where they do not engage exclusively in energetically expensive searching behaviour. These results exemplify the value of applying analytical approaches from the discipline of movement ecology to resolve fundamental behavioural questions.

The distinct movement behaviour associated with musth periods has the potential to allow automated detection of the reproductive state of GPS tracked male elephants. In particular, the three‐state HMM could automatically detect musth movement with high sensitivity and specificity in males over age 35. However, the lack of a distinctive difference between musth and non‐musth movements in males under age 35 means that the automated detection of musth in young males would be error‐prone. In addition, elephant movements can also be strongly influenced by other factors such as local and seasonal shifts in resource distributions, for example vegetation productivity (Boettiger et al., 2011; Bohrer et al., 2014), which means that the inclusion of geography and seasonality in detection algorithms may be important. Despite this, our results demonstrate that musth in older individuals can clearly be detected, which means that musth behaviour can potentially be studied directly from GPS tracking data for males with distinctive musth periods.

The results of our study exemplify the value of long‐term bio‐logging and applying analytical approaches from the discipline of movement ecology to resolve fundamental behavioural questions. The combination of long‐term visual observations and GPS tracking in this study has both revealed new insights into the reproductive behaviour of male elephants and demonstrates that musth behaviour can be remotely detected in elephants over 35 years old in the future without the need for field observation. Bio‐logging‐based assessments can also provide wider insights into life‐history strategies (Crossin, Cooke, Goldbogen, & Phillips, 2014; Harel et al., 2016; Hooten, Scharf, & Morales, 2018) and key factors structuring survival and, therefore, conservation status (Wilson, Wikelski, Wilson, & Cooke, 2015). The leveraging of bio‐logging to address intractable questions in ecological–evolutionary research is still in development, but promises to change understanding in a wide variety of disciplines (Kays et al., 2015; Nathan et al., 2008; Wilmers et al., 2015; Wilson et al., 2015).

The three‐state HMM developed for this study could be further utilized to identify distinctive changes in long‐term behaviour, such as migration, or to identify life‐history events, such as reproductive events. In elephants, given the advent of real‐time tracking (Wall et al., 2014), automatic detection of musth behaviour could be used to institute proactive management actions around musth bulls, such as enhancing protection of reproductively active males (e.g. avoiding legal hunting) or intervening when such males approach human‐dominated areas where associated heightened aggressive behaviours can be problematic. Musth detection can also serve to identify and protect important corridors for genetic transmission between subpopulations through human‐dominated landscapes, given the propensity for exploratory behaviour of reproductively active males, even when analysis is applied retrospectively. Moreover, the ability to detect musth periods directly from GPS tracking data will enable remote study of reproductive behaviour in elephants.

Overall, our results illustrate the potential of bio‐logging both for answering fundamental behavioural questions and for applied wildlife conservation and management. Specifically, our results indicate that movement‐related reproductive tactics change with age likely as a function of indeterminate body mass growth, which may have implications on life‐history parameters, as well as elephant conservation and population management. Older male elephants tend to be the target of both legal trophy hunting and illegal poaching. As reproductive allocation increases with age, removing dominant older males from the population could affect the reproductive dynamics in elephants by changing the dynamics of male–male competition (i.e. through reducing the density of dominant breeders). Such changes may alter the degree of reproductive skew in a population (driving changes in the effective population size), adjust the fitness benefits of allocation in movement relative to growth at different stages and lead to the selection of different physical characteristics (e.g. early maturity). Thus, human‐driven selection could drive fundamental changes to elephant reproductive tactics and life history, which could have lasting implications on elephant populations. The ability to detect reproductive behaviour directly from GPS tracking data means that elephant reproductive tactics can be remotely quantified, which can be used both to study elephant reproduction and to institute management strategies around the reproductive behaviour of this charismatic keystone species to help mitigate human–wildlife conflict.

## AUTHORS' CONTRIBUTIONS

L.A.T. and G.W. conceived the project; G.W. and I.D.‐H. collected the data; L.A.T. formatted the data; L.A.T., B.L. and D.L. analysed the data; L.A.T. led the writing of the manuscript. All authors contributed critically to the drafts and gave final approval for publication.

## Data Availability

A transposed version of the elephant GPS tracking data and the three‐state hidden Markov model code and an example model output are deposited in the Dryad Digital Repository: https://doi.org/10.5061/dryad.368cf90 (Taylor et al., 2019). The elephant GPS locations have been transposed from their original locations to protect the elephants.
