# Supplementary material for: Movement reveals reproductive tactics in male elephants
Source: J Anim Ecol. 2019 Jun 24;89(1):57–67. doi: 10.1111/1365-2656.13035 (PMC7004166; doi:10.1111/1365-2656.13035)
Supplement: Supplementary file 1 [file JANE-89-57-s001.pdf]

SUPPLEMENTARY MATERIALS

Movement reveals reproductive tactics in male elephants

Lucy A. Taylor, Fritz Vollrath, Ben Lambert, Daniel Lunn, Iain Douglas-Hamilton

& George Wittemyer

Supplementary Tables and Figures

Table S1. Total GPS tracking non-musth and musth days by month. Note the low number of musth observations between July and October.

**Month**	**Total GPS tracking days**	
	**Non-musth**	**Musth**
January	83	73
February	131	44
March	128	19
April	87	51
May	147	119
June	102	91
July	140	3
August	83	3
September	108	7
October	129	7
November	148	32
December	117	33
**Total**	1,403	482

Table S2. Model priors for three state-hidden Markov models.

Model	β (State 1, State 2, State 3)	σ (State 1, State 2, State 3)
Log-transformed daily mean speed	-1.0, 0.0, -1.0	0.75, 1.5, 0.75
Log-transformed 95% MCP	1.0, 2.0, 1.0	0.75, 1.5, 0.75

Table S3. Results of the linear mixed-effects models for log-transformed daily mean speed and log-transformed 95% MCP, and the binomial generalised linear mixed-effects model for the proportion of exploratory behaviour per day. Blank cells correspond to variables which were removed from the model and are therefore not significant. Note: Estimates for age are in years centred on a reference point of age 35, whereas the environmental covariates are standardised by removing the mean and dividing by the standard deviation. Marginal ($R^2_{LME(m)}$) and conditional R-squared values ($R^2_{LME(c)}$) were calculated using the methods described in Nakagawa and Schielzeth (2013).

Fixed-effect	Log-transformed speed (Estimate [95% CI])	Log-transformed 95% MCP (Estimate [95% CI])
(Intercept)	-1.03 [-1.16, -0.91]	1.08 [0.83, 1.33]
Musth	0.40 [0.31, 0.49]	0.84 [0.65, 1.02]
Age	-0.01 [-0.02, 0.00]	-0.02 [-0.04, 0.00]
Age^2		
NDVI	0.15 [0.08, 0.23]	0.30 [0.15, 0.45]
$NDVI^2$	-0.04 [-0.07, -0.01]	-0.07 [-0.14, 0.00]
Slope		
$Slope^2$	-0.01 [-0.02, -0.01]	-0.03 [-0.05, -0.01]
VRM	0.03 [0.01, 0.05]	0.05 [0.00, 0.1]
VRM^2	-0.03 [-0.05, -0.02]	-0.1 [-0.14, -0.07]
Water	0.07 [0.03, 0.11]	0.24 [0.15, 0.34]
$Water^2$	-0.01 [-0.01, 0.00]	-0.03 [-0.04, -0.02]
Protected	0.16 [0.11, 0.21]	0.36 [0.24, 0.49]
Musth * Age	0.02 [0.01, 0.03]	0.03 [0.00, 0.05]
Musth * Age^2		
Musth * NDVI		
Musth * $NDVI^2$	-0.07 [-0.12, -0.02]	-0.12 [-0.22, -0.02]
Musth * Slope		
Musth * $Slope^2$	0.01 [0.00, 0.02]	0.03 [0.00, 0.05]
Musth * VRM		
Musth * VRM^2		
Musth * Water		
Musth * $Water^2$		
Musth * Protected		
$R^2_{LME(m)}$	0.22	0.19
$R^2_{LME(c)}$	0.46	0.36

Table S4. Results of the three-state hidden Markov model of log-transformed daily mean speed (km h^{-1}) aiming to detect musth in male elephants. The number of observations corresponds to the number of visual observations correctly or incorrectly assigned by the three-state HMM. Note that the values for daily mean speed during the detected states includes all of the data within the detected time frames and thus contains both the false-positive and false-negative results.

Name	Age (years)	Three-state HMM		Musth observations							Daily mean speed (km h^{-1}) during detected state					
		Detected musth length (days)	SD of detected length (days)	Non-musth			Musth			Total % correct	Mean			Standard deviation		
				Correct	Incorrect	% Correct	Correct	Incorrect	% Correct		Non-musth	Musth	% Change	Non-musth	Musth	% Change
Nehru	28	17	23.6	9	0	100	0	1	0	90	0.37	0.38	3	0.14	0.14	-3
Edison	31	49	0.7	0	0	-	7	0	100	100	0.28	0.66	131	0.12	0.31	162
Theresai	33	30	28.9	4	1	80	3	0	100	88	0.42	0.50	19	0.23	0.20	-12
Winston	31	50	27.5	27	7	79	2	2	50	76	0.49	0.76	55	0.25	0.34	38
	32	30	33.1	15	7	68	0	2	0	63	0.42	0.43	3	0.26	0.25	-5
	32	86	2.4	5	9	36	8	0	100	59	0.34	0.56	66	0.11	0.23	116
	34	83	17.3	1	3	25	5	0	100	67	0.22	0.56	154	0.12	0.32	177
Apollo	38	13	10.9	10	0	100	1	0	100	100	0.53	0.73	38	0.21	0.16	-24
Esidai	38	93	13.7	18	4	82	17	0	100	90	0.32	0.61	92	0.16	0.23	48
	39	105	6.7	1	6	14	8	0	100	60	0.17	0.49	184	0.06	0.22	235
Kenyatta	42	72	0.0	15	0	100	8	0	100	100	0.34	0.85	151	0.20	0.36	77
	43	50	2.8	11	0	100	13	0	100	100	0.29	0.64	124	0.11	0.26	143
PrettyBomBom	44	67	1.7	18	1	95	12	3	80	88	0.33	0.62	87	0.15	0.21	36
Mungu	47	88	5.2	28	1	97	20	0	100	98	0.25	0.63	152	0.10	0.29	206
Matt	52	27	0.9	0	0	-	2	0	100	100	0.29	0.67	134	0.12	0.36	196
**Total count/ mean value**		51*	10.4*	162	39	84*	106	8	84*	90*	0.34*	0.61*	91*	0.15*	0.25*	89*
**Under 35**		40*	18.3*	61	27	77*	25	5	66*	86*	0.36*	0.53*	56*	0.17*	0.24*	57*
**Over 35**		59*	5.1*	101	12	88*	81	3	97*	94*	0.33*	0.66*	114*	0.14*	0.26*	111*

*After accounting for the effects of repeated individual

Table S5. Results of the three-state hidden Markov model of log-transformed 95% MCP aiming to detect musth in male elephants. The number of observations corresponds to the number of visual observations correctly or incorrectly assigned by the three-state HMM.

Name	Age (years)	Change point SD	Non-musth			Musth			Total % correct
			Correct	Incorrect	% Correct	Correct	Incorrect	% Correct	
Nehru	28	21.0	9	0	100	0	1	0	90
Edison	31	15.1	0	0		7	0	100	100
Theresai	33	37.5	4	1	80	3	0	100	88
Winston	31	20.0	16	18	47	4	0	100	53
	32	38.1	20	2	91	0	2	0	83
	32	4.3	8	6	57	7	1	88	68
	34	8.6	0	4	0	5	0	100	56
Apollo	38	64.4	8	2	80	1	0	100	82
Esidai	38	8.3	20	2	91	16	1	94	92
	39	7.3	2	5	29	8	0	100	67
Kenyatta	42	0.0	15	0	100	8	0	100	100
	43	3.5	11	0	100	13	0	100	100
PrettyBomBom	44	1.1	18	1	95	12	3	80	88
Mungu	47	4.3	28	1	97	20	0	100	98
Matt	52	1.4	0	0		2	0	100	100
**Total count/ mean value**		17.2*	159	42	82*	106	8	85*	89*
**Under 35**		22.8*	57	31	76*	26	4	68*	86*
**Over 35**		13.5*	102	11	86*	80	4	96*	91*

*After accounting for the effects of repeated individuals

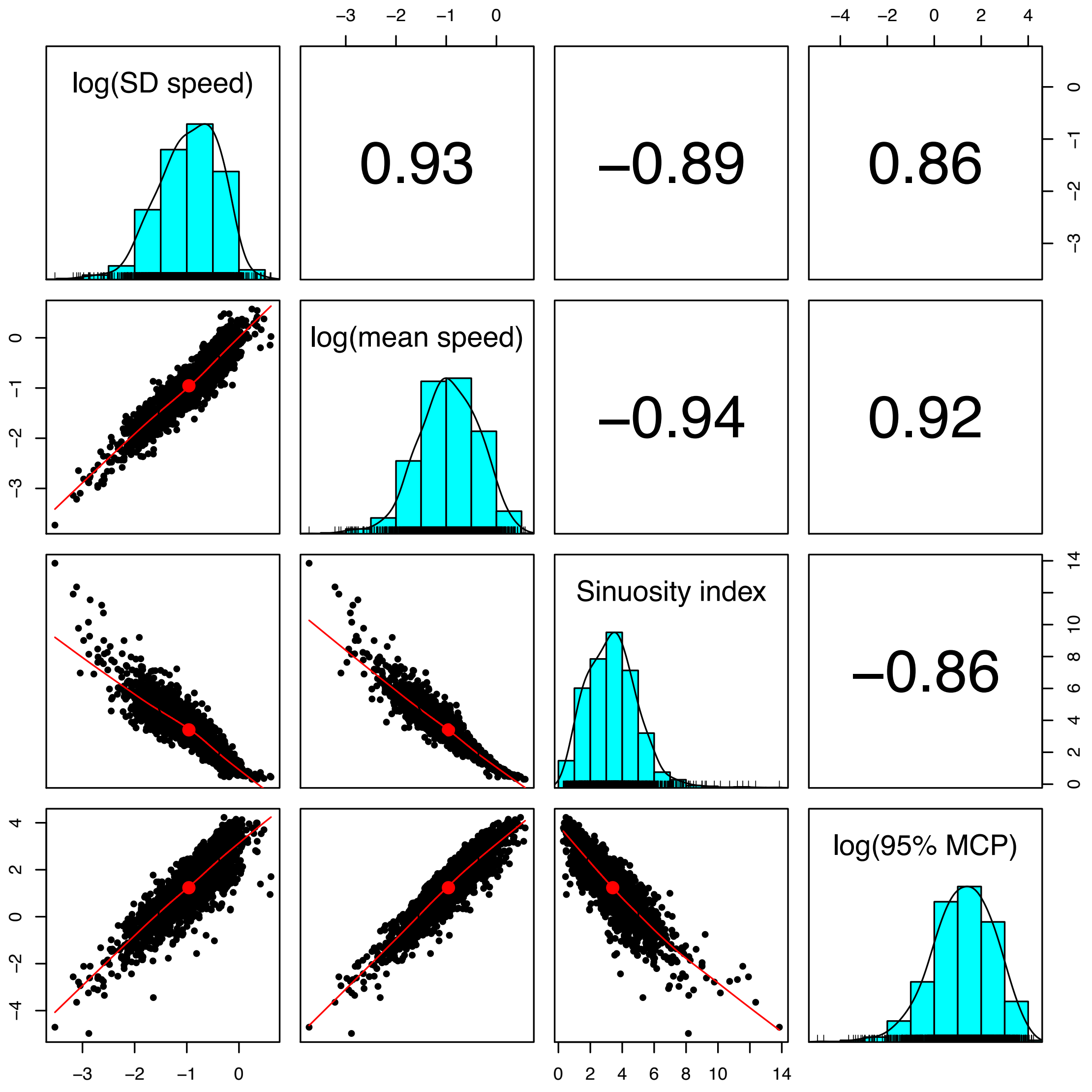

Figure S1. Relationship between log-transformed daily standard deviation of speed, log-transformed daily mean speed, sinuosity index, log-transformed 95% MCP and proportion of exploratory behaviour for the all GPS tracked days used in the linear mixed-effects model analysis.

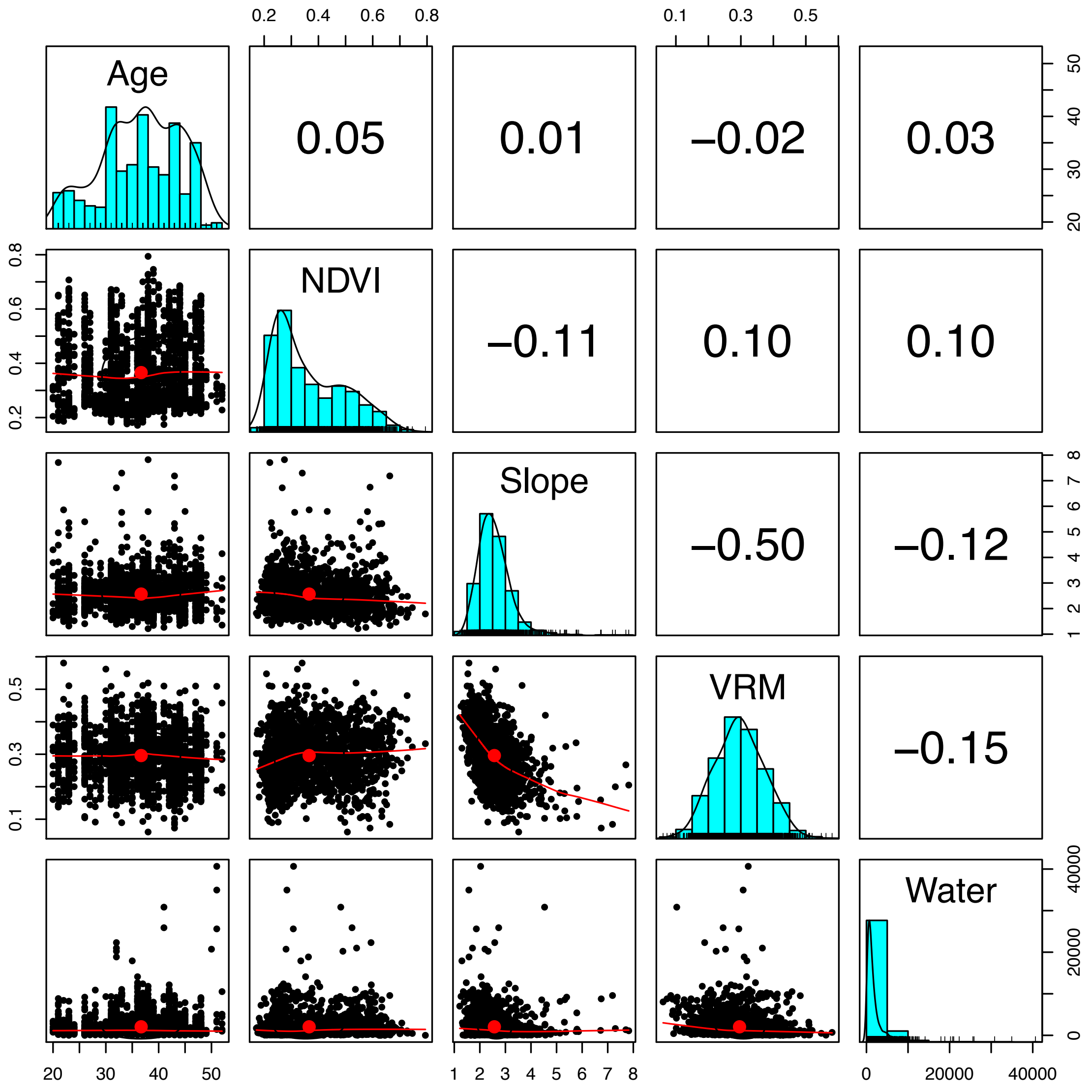

Figure S2. Relationship between all of the continuous covariates for the all GPS tracked days used in the linear mixed-effects model analysis. Covariates include the age at observation (years), daily mean NDVI, daily mean slope (degrees), daily mean vector ruggedness measure (VRM) and daily mean distance to water (km).

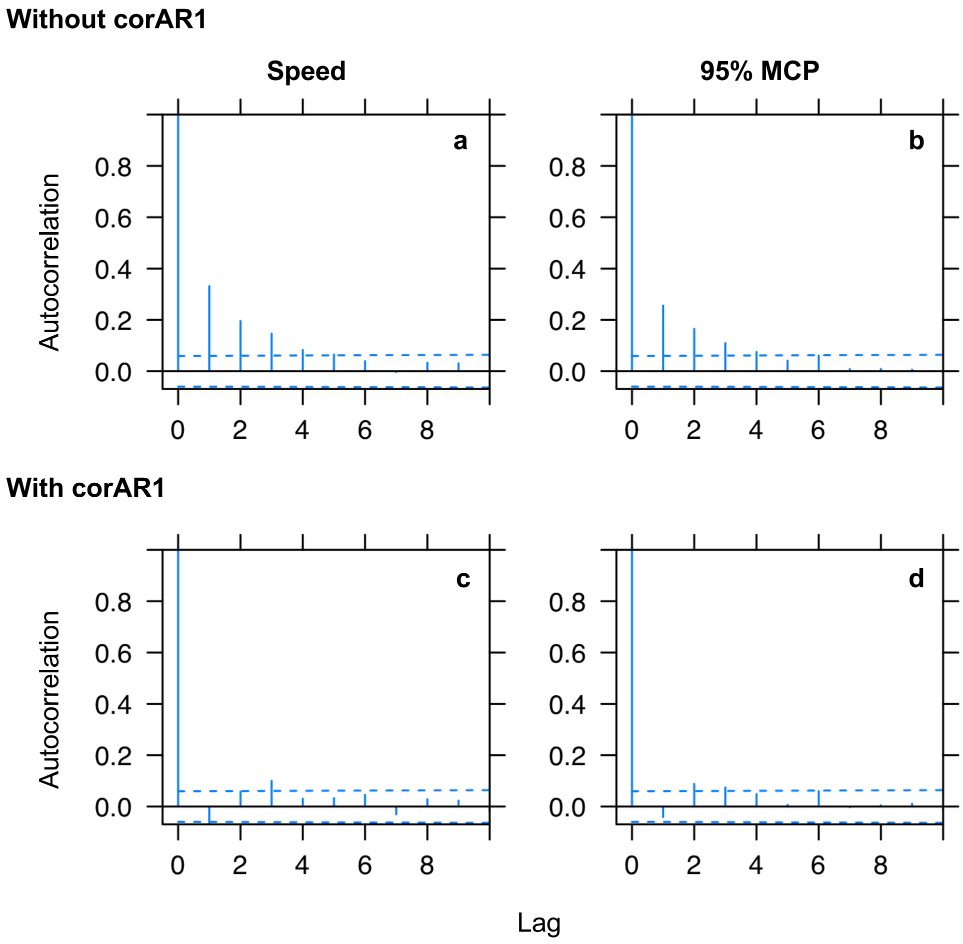

Figure S3. Autocorrelation of residuals (a-b) without and (c-d) with an autoregressive lag-1 correlation (corAR1) structure. Plots show the linear mixed-effects model of (a, c) log-transformed daily mean speed and (b, d) log-transformed 95% MCP. Note the autocorrelation in plots a-b, which is resolved by adding the corAR1 autocorrelation structure (c-d).

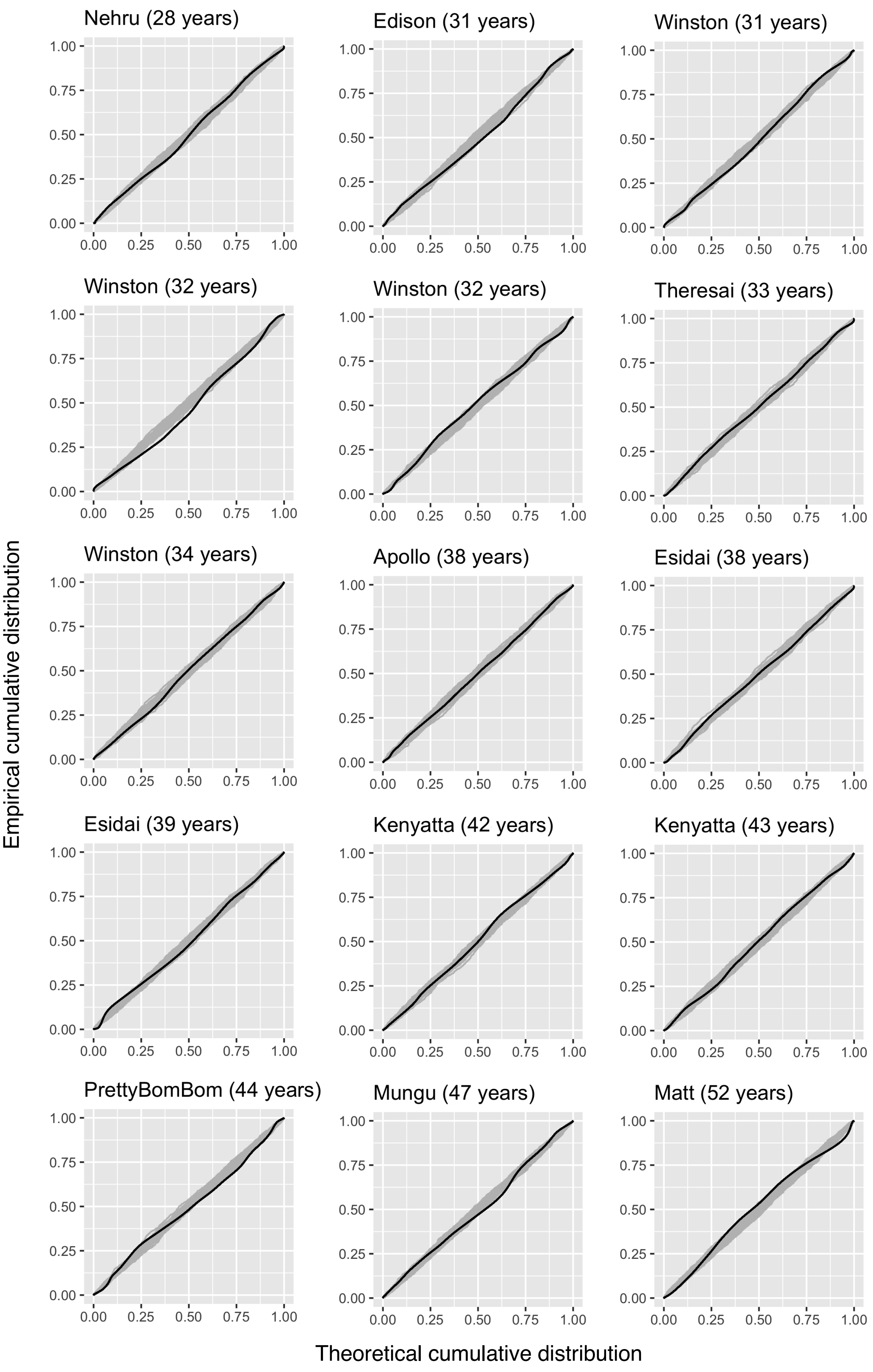

Figure S4. Posterior predictive checks of model fit of the three-state hidden Markov model for log-transformed daily mean speed aiming to detect musth periods in bull elephants.

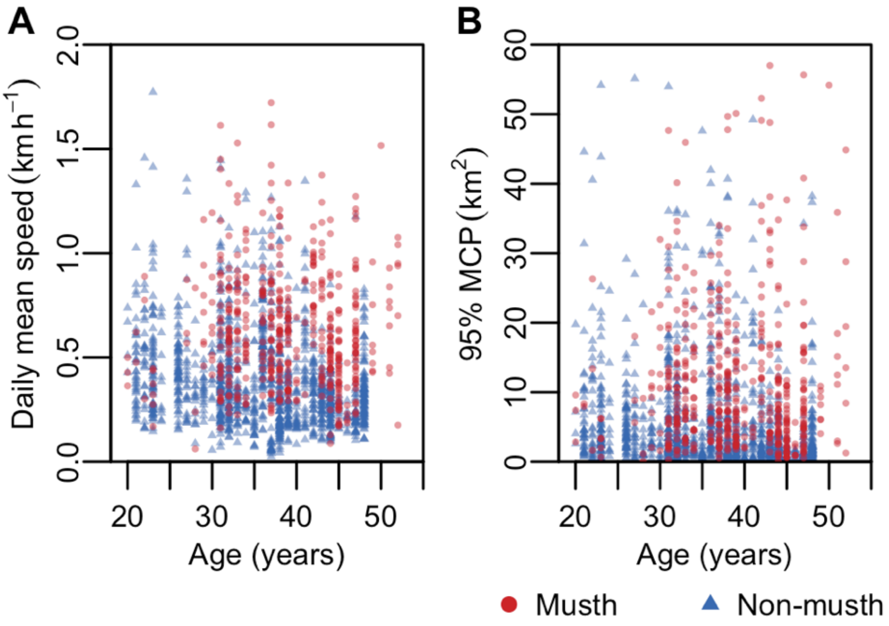

Figure S5. Raw data illustrating the relationship between daily mean speed (km h^{-1}) and (B) 95% MCP (km^2), and age (years) in musth (red) and non-musth (blue).

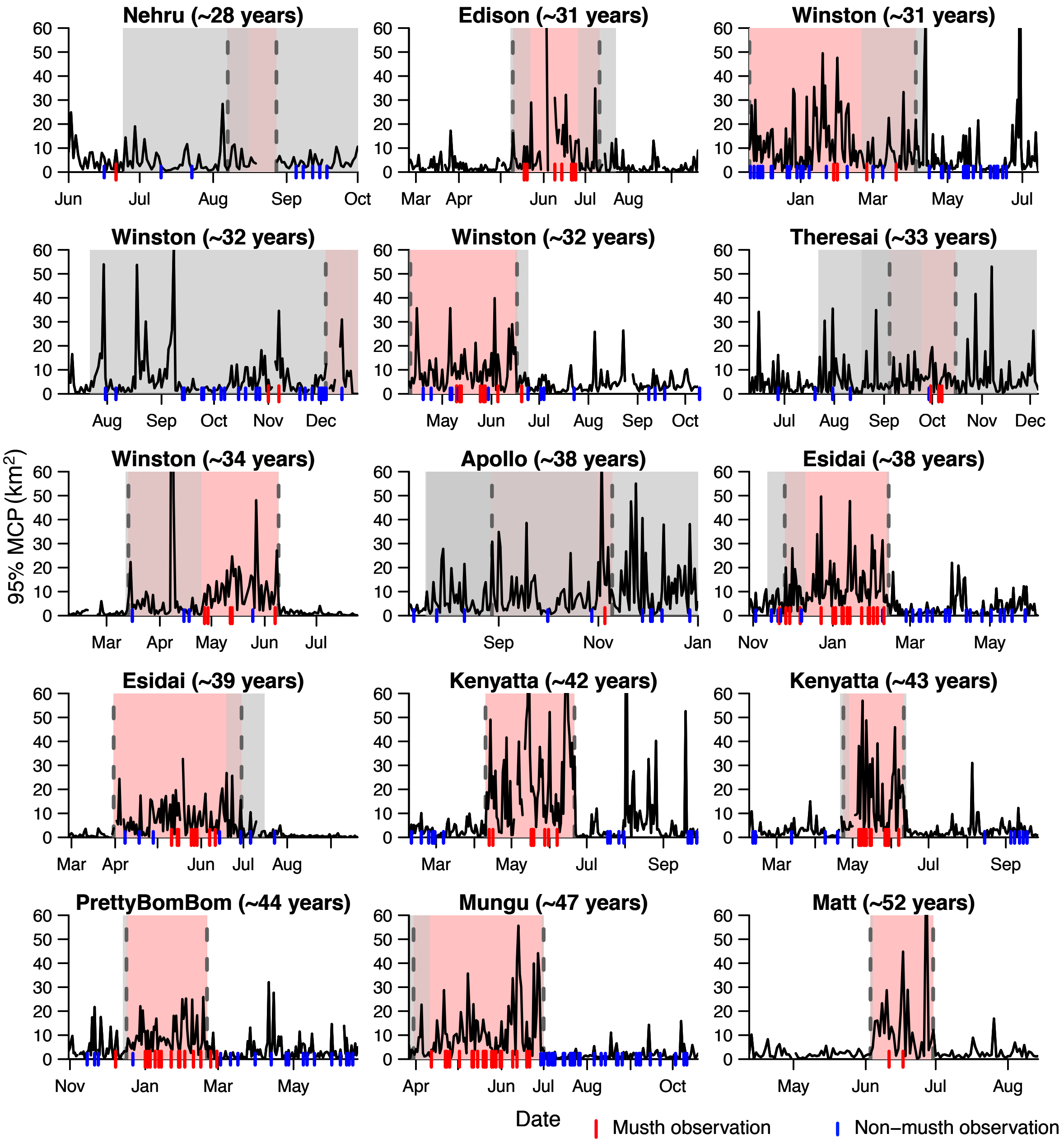

Figure S6. Three-state hidden Markov model results of the model for log-transformed 95% MCP aiming to detect musth periods in bull elephants. Plots show the untransformed 95% MCP (km^2) with the detected musth periods shaded in red. Grey shaded area indicates the corresponding credible interval (±95%). Visual observations of the bull in musth or non-musth are denoted by the red and blue lines at the base of the plot. Plots are ordered by age from youngest to oldest.
